# Uncovering the shared molecule and mechanism between ulcerative colitis and atherosclerosis: an integrative genomic analysis

**DOI:** 10.3389/fimmu.2023.1219457

**Published:** 2023-08-10

**Authors:** Jinke Huang, Fengyun Wang, Xudong Tang

**Affiliations:** ^1^ Department of Gastroenterology, Xiyuan Hospital of China Academy of Chinese Medical Sciences, Beijing, China; ^2^ Institute of Digestive Diseases, Xiyuan Hospital of China Academy of Chinese Medical Sciences, Beijing, China

**Keywords:** ulcerative colitis, atherosclerosis, co-occurrence, mechanism, PTPRC

## Abstract

**Background:**

Ulcerative colitis (UC) and atherosclerosis (AS) are closely related. However, the pathologic mechanisms underlying the co-occurrence of UC and AS are not well understood.

**Objects:**

To reveal the hub molecule and mechanism involved in the co-occurrence of UC and AS.

**Methods:**

Differentially expressed genes (DEGs) of UC and AS were obtained, and the shared DEGs of UC and AS were explored for biological function. Next, the hub genes were explored using the cytoHubba plugin. The predictive ability of the hub genes was measured by constructing the receiver operating characteristic curve. Analyses of immune infiltration and the single-gene gene set enrichment analysis (GSEA) for the hub genes were further carried out.

**Results:**

Identification of 59 DEGs (55 were upregulated and four were downregulated) shared by both UC and AS was performed. Enriched pathways of the shared DEGs were mainly related to immunity and inflammation. Protein tyrosine phosphatase, receptor type, C (PTPRC) was identified as the hub crosstalk gene for the comorbidity of UC and AS. The upregulation of PTPRC was correlated with mast cells resting, T cells CD4 memory resting, macrophages M0, and macrophages M1. Pathways of immune and inflammatory processes, including NF-kappa B, viral protein interaction with cytokine and cytokine receptor, and cytokine–cytokine receptor interaction, were significantly correlated with high expression of PTPRC in UC and AS.

**Conclusion:**

At the transcriptional level, our study reveals that imbalanced inflammatory and immune responses are the key pathological mechanisms underlying the comorbidity of UC and AS and that PTPRC is a key biomarker for the comorbidity of UC and AS.

## 1 Background

As an inflammatory bowel disease (IBD), ulcerative colitis (UC) is characterized by chronic inflammation originating in the rectum and extending proximally to the colon in a continuous manner (1). Recently, the incidence and prevalence of UC have increased globally, especially in developing countries (2). As a result of interactions between genetic factors, environmental factors, gut microbiology, and the immune system, patients with UC experience uncontrolled intestinal inflammation (3). However, the pathogenesis of UC has not yet been fully understood (4). As a common cause of death worldwide, atherosclerosis (AS) is a chronic inflammatory disease involving the arteries (5). A number of factors are considered to contribute to the accelerated development of AS, including chronic inflammation (6), autoimmune responses (7), and endothelial dysfunction (8). Ischemic cerebrovascular disease, ischemic heart disease, and peripheral arterial disease are the major forms of vascular disease, and AS is the leading cause of all these conditions (9).

Compared with the general population, patients with IBD exhibit a higher risk of early AS and increased intima–media thickness (10–13). Epidemiological data suggest that there is a significant increase in the incidence of arterial vascular events in IBD patients (14). Similarly, patients with AS also have an increased risk of developing IBD (15). The duration of UC was also found to be an independent risk factor for AS (16). These accumulating findings point to a strong link between UC and AS, although molecular mechanisms and pathological interactions remain unknown. Accordingly, there is undeniable clinical significance in investigating the molecular mechanisms linking UC and AS, as well as in identifying and treating these conditions at an early stage.

**Figure 1:**
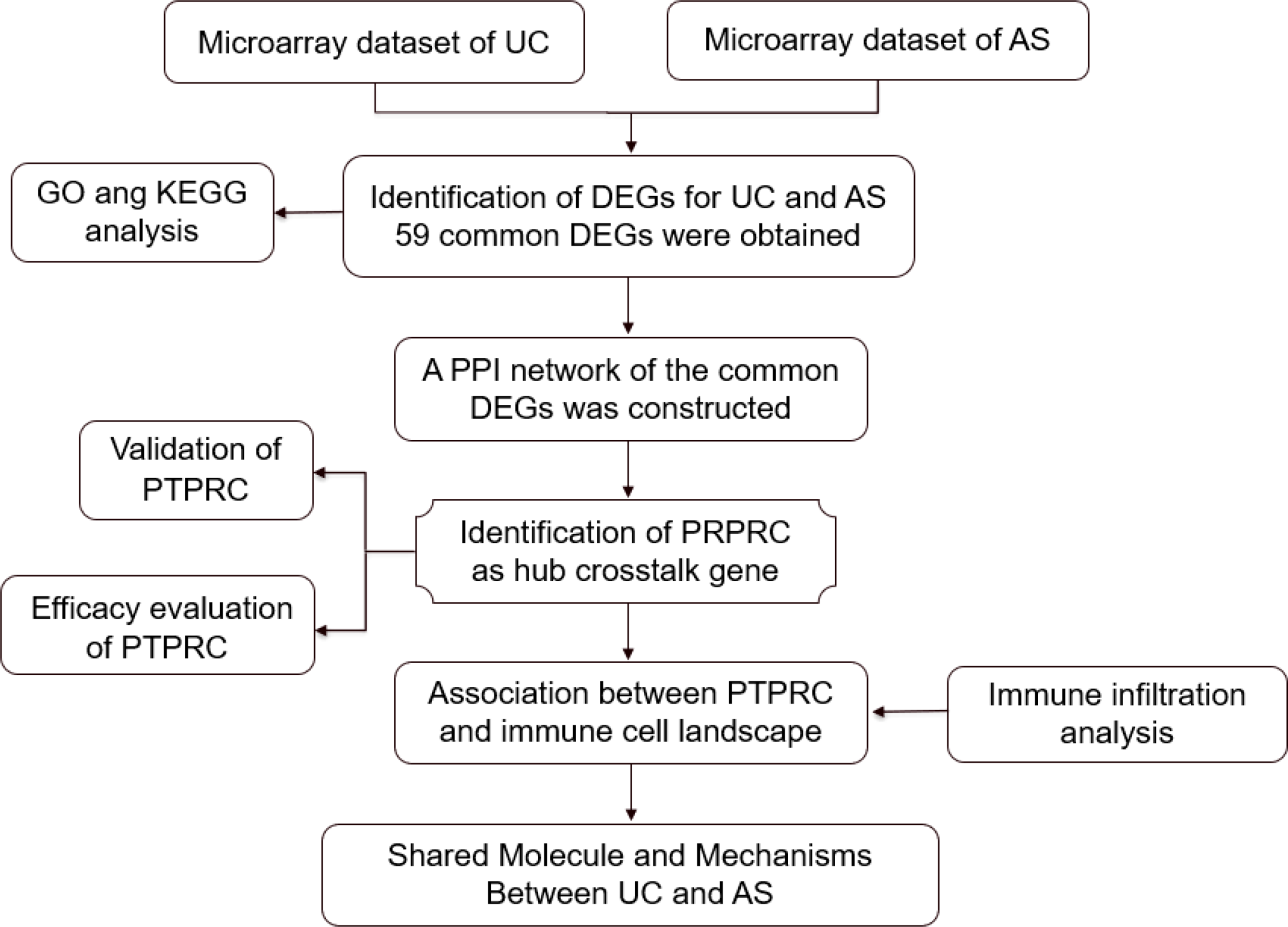
Research design flowchart.

In the current study, the transcriptome-level crosstalk mechanism between UC and AS was identified using microarray data (**Figure 1**). New insights into the biological mechanism of UC and AS were hypothesized to be provided by the crosstalk genes and associated pathways between the two diseases.

## 2 Materials and methods

### 2.1 Collection of microarray data

The following criteria were applied to retrieve microarray data of UC and AS from the Gene Expression Omnibus (GEO) database (www.ncbi.nlm.nih.gov/geo/): a) from the same sequencing platform, two distinct expression profiles were generated; b) the test specimens included should be from humans; c) no less than 10 samples should be included per group. Finally, four datasets numbered GSE92415 (17), GSE87473 (18), GSE100927 (19), and GSE28829 (20) were included in this study. GSE92415 contains 87 UC patients and 21 controls, GSE87473 contains 106 UC patients and 21 controls, GSE100927 contains 69 AS patients and 35 controls, and GSE28829 contains 13 early AS and 16 advanced AS. More details of the included datasets are listed in **Table 1**.

**Table 1:** Details on the included datasets.

Dataset	Platform	Samples	Disease	Group	Reference (PMID)
GSE92415	GPL13158	87 patients and 21 controls	UC	Discovery cohort	23735746
GSE100927	GPL17077	69 patients and 35 controls	AS	Discovery cohort	29500419
GSE87473	GPL13158	106 patients and 21 controls	UC	Validation cohort	29401083
GSE28829	GPL570	13 early AS and 16 advanced AS	AS	Validation cohort	22388324

AS, atherosclerosis; UC, ulcerative colitis.

### 2.2 Differentially expressed gene identification

The “limma” package in R software was used to determine the differentially expressed genes (DEGs) between the disease and control groups. Significant DEGs were thresholded at logFC (fold change)| ≥ 1 and *p*-value <0.05. In addition, the “VennDiagram” package was applied to locate the DEGs that were shared by UC and AS. The shared DEGs were saved for use in subsequent analyses.

### 2.3 Functional enrichment analysis

Gene Ontology (GO) and Kyoto Encyclopedia of Genes and Genomes (KEGG) enrichment analyses were performed using an online platform (Chinese version, https://www.bioinformatics.com.cn ; English version, http://www.bioinformatics.com.cn/srplot) that is based on the “clusterProfiler” and “pathview” packages. In this study, the Chinese version of this platform was used to obtain visual results of enrichment analysis after inputting the common DEGs and selecting species as human.

### 2.4 Construction of PPI network and identification of hub genes

The construction of the protein–protein interaction (PPI) network was carried out based on the STRING database (https://cn.string-db.org/). Cytoscape (version 3.7.2) was used to visualize the PPI network. Subsequently, the hub genes were analyzed using the cytoHubba plug-in of Cytoscape based on 10 common algorithms (MNC, MCC, EPC, Degree, Betweenness, Closeness, Radiality, BottleNeck EcCentricity, and Stress).

### 2.5 Hub gene expression validation

To improve confidence, the mRNA expression of identified hub genes was verified in GSE87473 and GSE28829. An independent t-test was applied to compare the hub gene expression between the cases and controls. *p*-Value <0.05 was considered significant

### 2.6 Efficacy evaluation of hub genes

Receiver operating characteristic (ROC) curves were performed using the “pROC” package to evaluate the predictive accuracy of the hub genes. Discrimination is absent if the area under the curve (AUC) is less than 0.5, adequate if it is between 0.7 and 0.8, excellent if it is between 0.8 and 0.9, and superb if it is greater than 0.9 (21).

### 2.7 Assessment of the immune landscape

With the use of the computational strategy of CIBERSORT (http://cibersort.stanford.edu/), the expression levels of immune cells for each sample of GSE92415 and GSE100927 were quantified. Next, Spearman’s approach was used to evaluate the association between the expression of the hub genes and the expression of immune cells in case group samples from the datasets.

### 2.8 GSEA of hub genes

Based on a single hub gene expression level, samples of the dataset were initially categorized as high expression or low expression. Subsequently, gene set enrichment analysis (GSEA) was performed using the “GSVA” package to reveal the pathways involved in the hub genes. Q value <0.25, *p* < 0.05, and |normalized enrichment score (NES)| > 1 were used as the criteria for enrichment analysis.

## 3 Results

### 3.1 Differential gene screening

**Figure 2:**
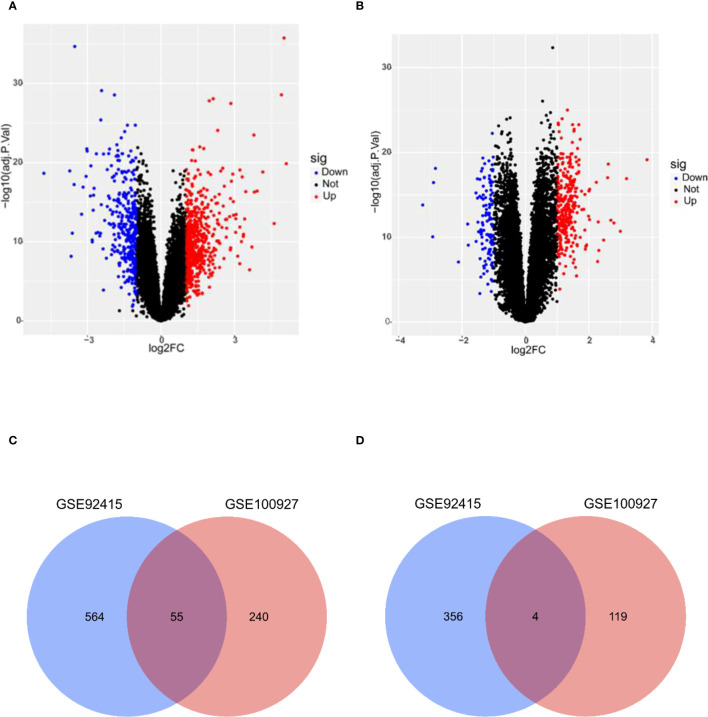
**(A)** DEGs shown on a volcano plot in GSE92415. **(B)** DEGs shown on a volcano plot in GSE100927. **(C)** Venn diagram of upregulated DEGs shared by UC and AS. **(D)** Venn diagram of downregulated DEGs shared by UC and AS. DEGs, differentially expressed genes; UC, ulcerative colitis; AS, atherosclerosis.

A total of 979 DEGs for UC (**Figure 2A**) and 418 DEGs for AS (**Figure 2B**) were obtained from GSE92415 and GSE100927, respectively. After the overlapping analysis, 59 shared DEGs of UC and AS were identified, of which four were downregulated genes and 55 were upregulated genes (**Figures 2C, D**).

### 3.2 Functional enrichment analysis

**Figure 3:**
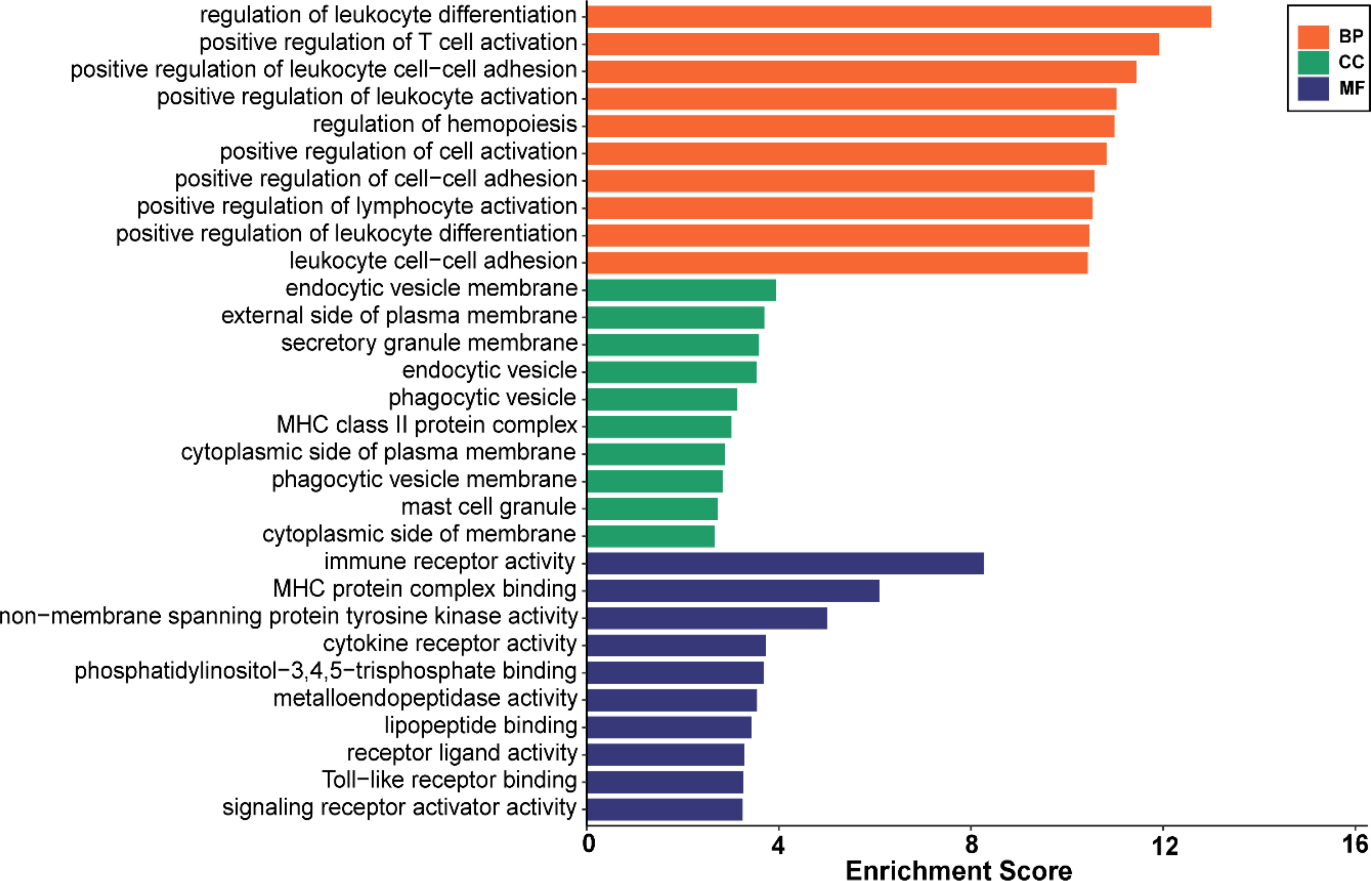
The enrichment analysis results of GO terms. GO, Gene Ontology.

**Figure 4:**
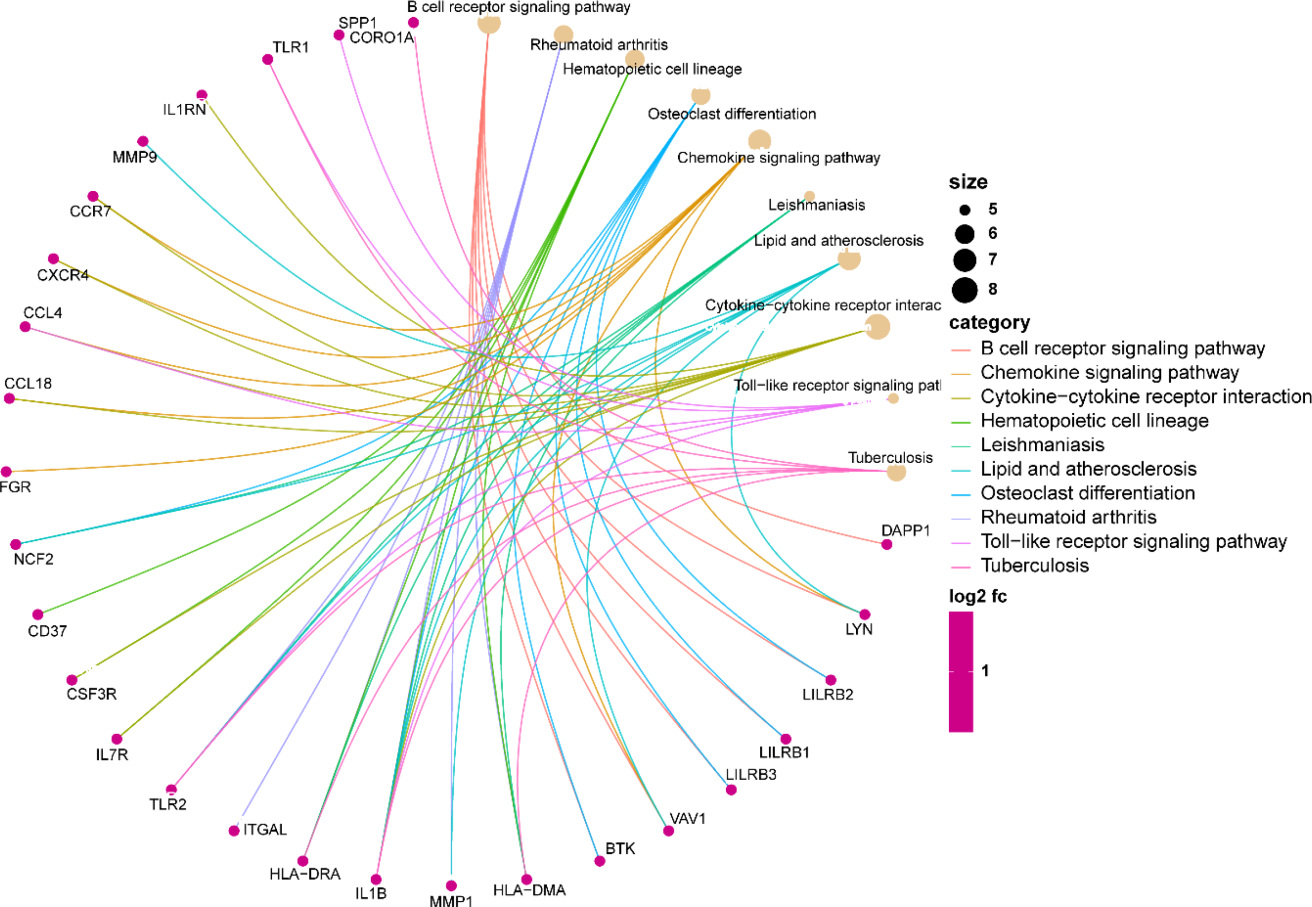
The enrichment analysis results of KEGG. KEGG, Kyoto Encyclopedia of Genes and Genomes.

Analysis of GO enrichment revealed that the shared DEGs were primarily enriched in the regulation of leukocyte differentiation, positive regulation of leukocyte cell–cell adhesion, and positive regulation of T-cell activation (**Figure 3**). In terms of the KEGG pathway, the significant enrichment pathways were rheumatoid arthritis, hematopoietic cell lineage, B-cell receptor signaling pathway, osteoclast differentiation, and chemokine signaling pathway (**Figure 4**). Interestingly, the shared DEGs were primarily involved in pathways related to immunity and inflammation.

### 3.3 Construction of PPI network and identification of hub crosstalk gene

**Figure 5:**
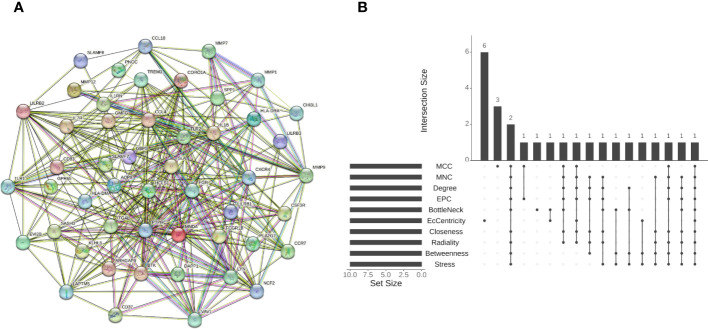
**(A)** STRING-based PPI network of shared DEGs. **(B)** Ten algorithms to screen hub genes by R package “UpSet”. PPI, protein–protein interaction; DEGs, differentially expressed genes.

The PPI network of the shared DEGs contained 50 nodes and 550 edges, with interaction scores greater than 0.400 (**Figure 5A**). Cytoscape plug-in cytoHubba was utilized to obtain the hub genes, and the 10 algorithms all pointed to protein tyrosine phosphatase, receptor type, C (PTPRC) as the hub crosstalk gene between UC and AS (**Figure 5B**; **Table 2**).

**Table 2:** The top 10 hub genes rank in cytoHubba.

Rank	MCC	MNC	Degree	EPC	BottleNeck	EcCentricity	Closeness	Radiality	Betweenness	Stress
1	PTPRC	PTPRC	PTPRC	PTPRC	PTPRC	PTPRC	PTPRC	PTPRC	PTPRC	PTPRC
2	IL1B	IL1B	IL1B	IL1B	MMP9	GPR65	IL1B	IL1B	IL1B	IL1B
3	TLR2	TLR2	TLR2	TLR2	VAV1	LYN	TLR2	TLR2	LILRB2	MMP9
4	CCL4	FGR	FGR	FGR	SPP1	VAV1	FGR	LILRB2	MMP9	TLR2
5	TLR1	LILRB2	LILRB2	CCL4	LILRB2	CCL4	LILRB2	FGR	FGR	FGR
6	NCF2	BTK	BTK	BTK	BTK	CCR7	BTK	CCL4	TLR2	LILRB2
7	TREM1	CCL4	CCL4	TLR1	TLR1	KLHL6	CCL4	MNDA	HLA-DRA	HLA-DRA
8	ITGAL	CXCR4	MMP9	LILRB2	HLA-DRA	FCGR1B	MNDA	BTK	MNDA	CXCR4
9	LILRB2	MNDA	MNDA	MNDA	IL1B	SLAMF8	TLR1	TLR1	CXCR4	BTK
10	CD83	TLR1	TLR1	NCF2	FGR	IL7R	CXCR4	CXCR4	LYN	LYN

### 3.4 Validation of PTPRC

**Figure 6:**
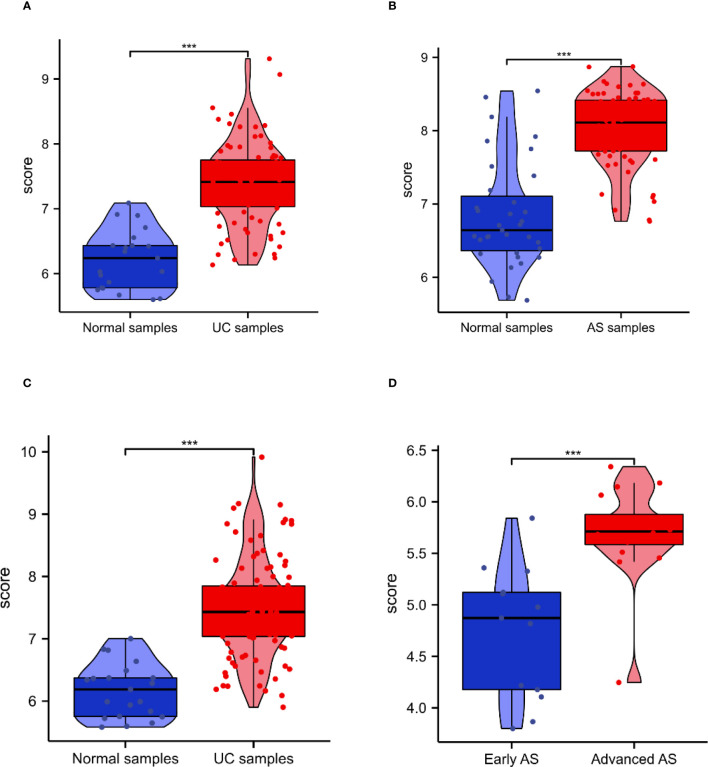
The expression and diagnosis significance of PTPRC in UC and AS. **(A)** PTPRC expression levels in UC patients were determined using GSE92415. **(B)** PTPRC expression levels in AS patients were determined using GSE92415. **(C)** PTPRC expression levels in UC patients were validated using GSE87473. **(D)** PTPRC expression levels in AS patients were validated using GSE28829. UC, ulcerative colitis; AS, atherosclerosis. ***P < 0.0005.

The expression of PTPRC was higher in both UC and AS cases than in controls (**Figures 6A, B**). GSE87473 was used for UC validation, and GSE28829 for AS validation. Interestingly, PTPRC was significantly upregulated in UC compared to controls, and PTPRC was also significantly upregulated in advanced AS compared to early AS (**Figures 6C, D**).

### 3.5 Efficacy evaluation of PTPRC

**Figure 7:**
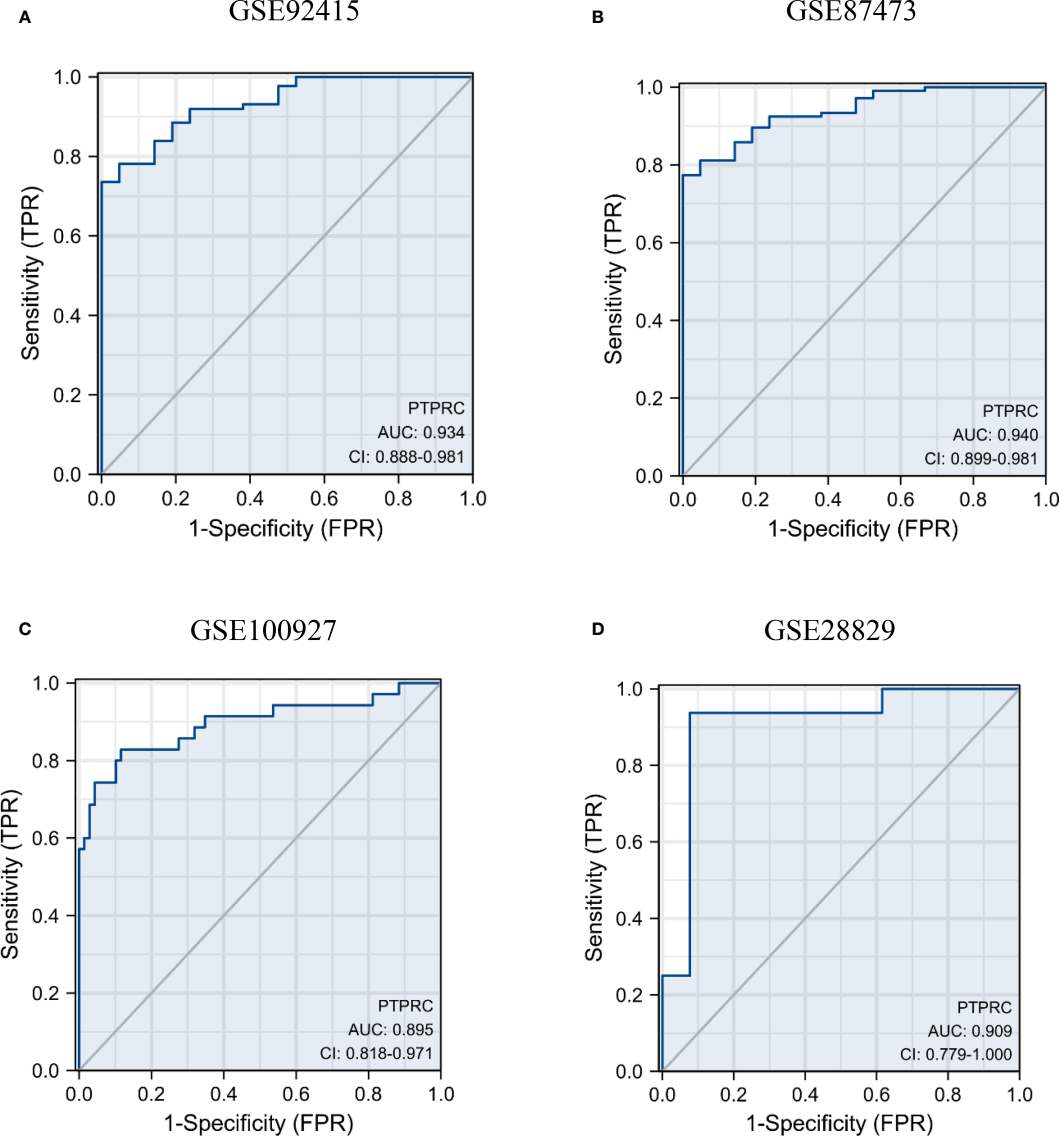
Diagnosis significance of PTPRC in UC and AS. Diagnostic efficacy of PTPRC for UC **(A, B)** and AS was analyzed using ROC curves **(C, D)**. UC, ulcerative colitis; AS, atherosclerosis; ROC, receiver operating characteristic.

The results of the efficacy evaluation of PTPRC revealed that the AUC values were both greater than 0.9 in GSE92415 and GSE100927 (**Figures 7A, B**). Further ROC analyses of the external UC and AS datasets were conducted to confirm the diagnostic performance, and the results showed that PTPRC had high predictive accuracy (**Figures 7C, D**).

### 3.6 Association between PTPRC and immune cell landscape

**Figure 8:**
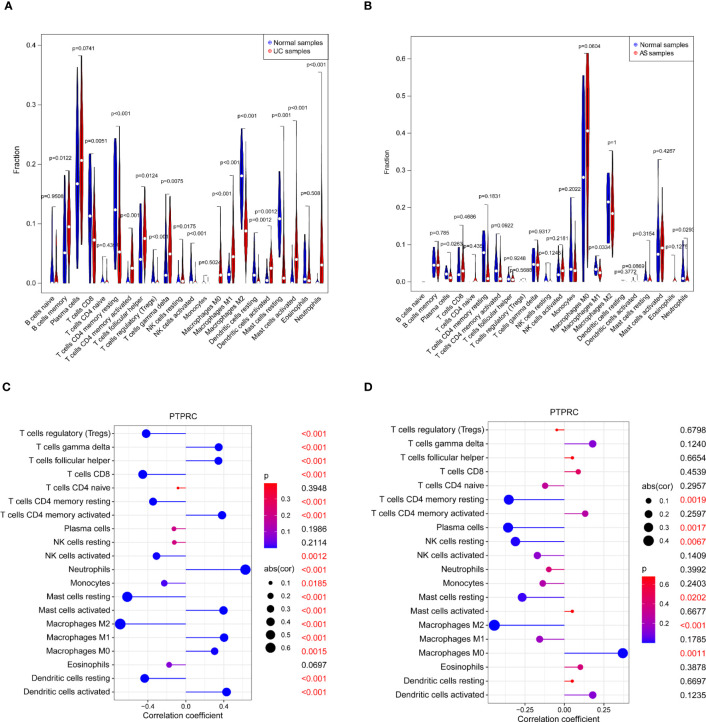
**(A)** The immune infiltration landscape in UC. **(B)** The immune infiltration landscape in AS. **(C)** Association between PTPRC and immune infiltrating cells in UC. **(D)** Association between PTPRC and immune infiltrating cells in AS. UC, ulcerative colitis; AS, atherosclerosis.

In comparison to the control group, the immune landscape in UC and AS was significantly altered, according to the results of the CIBERSORT algorithm (**Figures 8A, B**). Furthermore, correlation analysis suggested that PTPRC was significantly corrected with the expression of mast cells resting, T cells CD4 memory resting, macrophages M1, and macrophages M0 (**Figures 8C, D**).

### 3.7 GSEA of PTPRC

**Figure 9:**
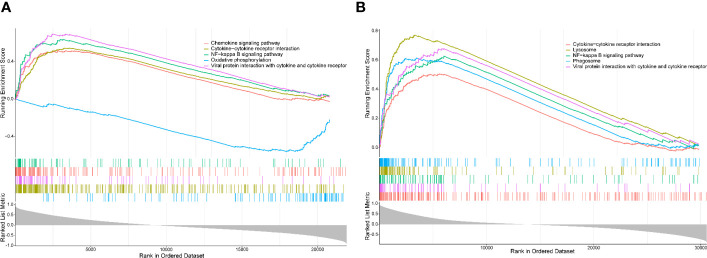
**(A)** Single-gene GSEA for PTPRC in UC using GSE92415. **(B)** Single-gene GSEA for PTPRC in AS using GSE100927. GSEA, gene set enrichment analysis; UC, ulcerative colitis; AS, atherosclerosis.

The single-gene (PTPRC) GSEA in UC showed markedly enriched pathways including NF-kappa B, cytokine–cytokine receptor interaction, oxidative phosphorylation, and chemokine signaling pathway (**Figure 9A**), while NF-kappa B, cytokine–cytokine receptor interaction, lysosome, and phagosome were identified in AS (**Figure 9B**). Interestingly, almost all pathways identified in UC and AS were related to immunity and inflammation.

## 4 Discussion

The correlation between UC and AS is continuously being revealed. It has been reported that UC patients are significantly more likely to develop AS than the general population (10–13). The most frequent cause of death in AS patients is cardiovascular and cerebrovascular disease (9), and patients with IBD have a higher risk of cardiac and cerebrovascular events when compared to matched non-IBD subjects (22, 23). Furthermore, the risk of IBD is increased in patients with AS when compared with matched non-AS subjects (15). In recent years, numerous candidate genetic loci have been identified. However, few studies concentrate on the genetic mechanism and biomarkers that are shared. In this study, 59 shared genes of UC and AS were identified, and the enriched biological functions and enriched pathways of these genes were mainly related to immunity and inflammation. Further exploration suggested that PTPRC was the hub crosstalk gene of the comorbidity of UC and AS, and PTPRC played a regulatory role in the comorbidity of UC and AS mainly through immune inflammation-related pathways.

The KEGG enrichment analysis revealed that the shared DEGs between UC and AS showed significant enrichment for rheumatoid arthritis, hematopoietic cell lineage, B-cell receptor signaling pathway, osteoclast differentiation, and chemokine signaling pathway. A crucial role for the B-cell receptor signaling pathway is played by numerous systemic autoimmune disorders. In patients with UC, significant abnormalities in the B-cell receptor signaling pathway were observed (24). AS is a lipid-driven vascular inflammatory disease, and the effects of B cells on AS have been widely recognized (25). Abnormalities in the B-cell signaling pathway are critical to the development of AS, and regulating B-cell preference pathways may be a promising strategy to prevent AS (25). Macrophages induced by the chemokine enter the wall of the blood vessels, which is a key pathological characteristic of the progression of AS (26). Chemokine receptor signaling can promote or prevent UC depending on the receptor. The balance of chemokines and their receptors is fraught with potential for inflammatory modulation in UC, and their promise as therapeutic molecular targets is widely recognized (27). Similarly, chemokine receptor signaling can influence the pathological progression of AS depending on the receptor. Several drugs created based on these concepts have shown positive benefits in animal models (28). Furthermore, as a key pathway in immunomodulation, rheumatoid arthritis possesses similar roles and therapeutic potential in UC and AS. Interestingly, these functional enrichment findings point to immune and inflammatory pathways as potential contributors to the co-occurrence of UC and AS. IBD is considered an immune-mediated disease causing systemic vascular inflammation. Immune system dysregulation, abnormal platelet function, and endothelial dysfunction all contribute to AS (29, 30). Increasing evidence suggests that autoimmune-related chronic inflammation is present in both IBD and AS (31). Furthermore, the overlap of IBD with AS treatment has also been reported. According to a retrospective matched case–control study, the use of statin was corrected with a lower risk of UC (OR 0.70, 0.65–0.76) (32). Patients who received 5-ASA experienced a decrease in their risk of developing ischemic heart disease, according to a nationwide, population-based retrospective cohort study (33). A multicenter prospective longitudinal study found a significant improvement in arterial pulse wave velocity in IBD patients after receiving long-term anti-TNF therapy, indicating that decreased inflammation improves endothelial dysfunction (34). In addition, mice with colitis treated with clopidogrel showed improvements in disease activity and colonic mucosal damage (35). Therefore, abnormal immune and inflammatory responses are the mechanisms of comorbidity of UC and AS, and comprehensive adjustment of immune and response status is the basis for the combined prevention and treatment of these two diseases.

PTPRC was identified as the hub crosstalk gene between UC and AS according to the cytoHubba plug-in of Cytoscape. PTPRC, a significant leukocyte antigen, has been demonstrated to be a crucial regulator of T- and B-cell antigen receptor signaling and to have played a crucial role in the innate immune system (36). According to the results of the present study, the expression levels of PTPRC were significantly higher in UC and AS patients, and this gene also showed excellent discriminatory power for these two diseases. Importantly, these findings were also confirmed by the external data. It has been reported that PTPRC is overexpressed in the serum of patients with IBD (37). Furthermore, in the trinitrobenzene sulfonic acid-induced colitis model, increased immunoreactivity of PTPRC was detected (38). However, in PTPRC knockout mice, colitis follows dietary changes (39). For AS, PTPRC has been uncovered as a hub gene (40–43), and it also has been experimentally confirmed by animal models (44). Inflammatory and immune pathways were involved in the comorbidity of UC and AS, according to enrichment analysis of the shared DEGs. Immune infiltration analysis further revealed that the immune landscape in UC and AS was significantly different from the control group. Additionally, correlation analysis revealed a significant relationship between PTPRC and the expression of mast cells resting, T cells CD4 memory resting, macrophages M0, and macrophages M1. As a result of the single-gene GSEA, NF-kappa B, cytokine–cytokine receptor interaction, and viral protein interaction with cytokine and cytokine receptors were identified. The above results all imply that PTPRC may mediate the comorbidity of UC and AS by regulating immunity and inflammation through the NF-kappa B, cytokine–cytokine receptor interaction, and viral protein interaction with cytokine and cytokine receptors. For the early detection and prevention of UC and AS comorbidity, PTPRC may be a potential biomarker.

Limitations should be acknowledged. First, we did not identify microarray data from patients with comorbidities of UC and AS; therefore, the dataset used in this study were either UC or AS, which may have caused the hub molecule and mechanism we have uncovered for the comorbidity of UC and AS to be an over-interpretation of the data. Second, we were unable to gather enough clinical samples of UC and AS within a limited timeframe. Thus, even if the external microarray datasets validated the results of bioinformatics analysis, their reproducibility and general application still need to be validated with clinical samples in the future. Third, the function of the hub genes must be validated *in vitro*, which will be the focus of our future research.

## 5 Conclusion

At the transcriptional level, our study reveals that imbalanced inflammatory and immune responses are the key pathological mechanisms underlying the comorbidity of UC and AS and that PTPRC is a key biomarker for the comorbidity of UC and AS.

## Data availability statement

The original contributions presented in the study are included in the article/supplementary material, further inquiries can be directed to the corresponding author.

## Author contributions

JH drafted the manuscript. FW helped with implementation of this work. XT contributed to the methodology, review, and editing of the manuscript. All authors read and approved the final manuscript.
